# *'I just keep thinking I haven't got it because I'm not yellow'*: a qualitative study of the factors that influence the uptake of Hepatitis C testing by prisoners

**DOI:** 10.1186/1471-2458-7-98

**Published:** 2007-06-07

**Authors:** Fu-Meng Khaw, Lynne Stobbart, Madeleine J Murtagh

**Affiliations:** 1 North East Health Protection Unit, Health Protection Agency, Institute of Pathology, Newcastle General Hospital, Westgate Road, Newcastle upon Tyne NE4 6BE, UK; 2 Institute of Health and Society, Newcastle University, Framlington Place, Newcastle upon Tyne NE2 4HH, UK

## Abstract

**Background:**

Hepatitis C viral (HCV) infection is a significant public health problem. In the UK, an estimated 200,000 individuals have active HCV infection, most of whom are injecting drug users (IDUs). Many IDUs spend time within the prison system therefore screening for HCV infection in this setting is important. However, uptake of testing within prisons is very low.

**Methods:**

Qualitative interview study. 30 interviews with 25 male and 5 female prisoners with a history of injecting drug use.

**Results:**

Personal and institutional barriers to uptake of testing for HCV were identified. Personal barriers included: prisoners' fears and lack of knowledge about HCV, low motivation for testing, lack of awareness about the testing procedure, and concerns about confidentiality and stigma. Institutional barriers included: the prisons' applications procedure for testing, inadequate pre- and post-test discussion, lack of pro-active approaches to offering testing, and lack of continuity of care on discharge and transfer.

**Conclusion:**

This study highlights potential areas of development in the management of HCV in prisons. Further research is needed to evaluate care pathways for HCV in the prison setting and to develop and assess interventions to improve the uptake of testing for HCV by prisoners.

## Background

Hepatitis C viral (HCV) infection is a significant public health problem. Chronic infection, if left untreated, can lead to serious illness in the long-term such as cirrhosis and cancer of the liver. In England, the estimated prevalence of active HCV infection is 0.4%, equating to 200,000 cases[1]. The overwhelming burden of disease is associated with injecting drug use (IDU), which is identified as a risk factor in over 80% of cases[2]. Amongst IDUs, HCV prevalence rates of 40–80% have been reported[3,4]. The custodial setting has a high proportion of prisoners with a history of IDU; estimated at around 24%[5]. Up to 61% of IDUs have spent time within the prison system[4].

The Hepatitis C Strategy for England, 2002, encourages testing for HCV in high risk populations[1]. There is good evidence that selective testing for HCV in IDUs, is effective in the long-term[6]. A recent study demonstrated the cost-effectiveness of screening for HCV in the prison population[7]. Whilst in prison, IDUs lead a less chaotic life than they would in the community. Hence, the prison setting is an important environment to offer health promotion and treatment interventions.

Although HCV testing is available in the prison healthcare system, the uptake of such opportunities is low; Skipper et al reported an uptake rate amongst prisoners of only 8.5%[8]. The reasons for this low uptake are not known. The purpose of this study was to identify the factors that influence the uptake of testing for HCV infection by prisoners. Knowledge of these barriers will help to optimise the ways in which testing opportunities are made available to prisoners.

## Methods

This was a qualitative study using audio-recorded semi-structured interviews in a sample of purposively selected prisoners. Participants were recruited from three prisons in Northeast England: HMP Durham; HMP Frankland and HMP Low Newton (Table 1). Prisoners were eligible if they were aged 18 years or over, had injected drugs in the past and were able to communicate in English. At HMP Frankland prisoners with a history of injecting drug use were identified from referrals to the Counselling, Assessment, Referral, Advice and Throughcare Service (CARATS). Eligible participants at HMP Durham were identified from referrals to the detoxification service. At HMP Low Newton participants were identified from data obtained from the Grubin Tool health assessment completed on reception.

**Table 1:** Characteristics of participating prisons

**Prison**	**Geographic area**	**Gender of inmates**	**Typical duration of stay**	Category*	**Capacity**
HMP Durham	Local	Male	Short	B	919
HMP Frankland	National	Male	Long	A	720
HMP YOI Low Newton	Local	Female	Short	A	396

* A Category A Prison is a maximum security prison, highly secured, and used mainly for high risk offenders, whose escape would be highly dangerous to the public or the police or the security of the state, no matter how unlikely that escape might be, and for whom the aim must be to make escape impossible. A Category B Prison is for prisoners for whom the very highest conditions of security are not necessary, but for whom escape must be made very difficult [15]

The interview schedule was developed through consultation with a project steering group, comprising prison healthcare staff, specialists in communicable disease control, and representatives from Primary Care Trusts and Drug and Alcohol Action Teams. Prisoners were provided with information sheets about the study and asked to give written consent to participate in the study prior to interview. Information and consent documents made clear that participation or non-participation in the study would not prejudice nor enhance their treatment in the prison. No reimbursement or inducements were offered to prisoners.

All prisoners were interviewed by LS in the health care facility at each establishment. LS was accompanied by a CARATS worker who acted as advocate for the prisoner. Interviews lasted between 10 and 40 minutes. All interviews were audio-recorded (with the prisoner's consent), transcribed, and then checked for accuracy against the recording. The research team (LS, MJM, FMK) analysed transcripts using established procedures of constant comparative analysis[9] from which themes were developed from the participants' responses.

The study was reviewed and given a favourable opinion by the Northern and Yorkshire Multi-centre Research Ethics Committee (MREC number: 04MRE0384). The study was approved by the Prisons Research Applications and Ethics Panel (PRAEP).

## Results

Ninety-nine eligible prisoners were identified. Fifty-five declined to participate. Forty-four initially agreed to participate but of these 10 declined to participate on the day, three were transferred to another institution prior to interview and one was held in a segregation unit during the available interview period. 30 prisoners took part in the study; 25 male and five female. Table 2 demonstrates that 19 participants had already taken up opportunities for HCV testing, most whilst in the prison system.

**Table 2:** Uptake of testing opportunities by study participants

Prison (total participants)	Testing status	Venue of testing*	No. with a positive test result
HMP Durham (12)	9 tested	Prison 5	2
	1 applied, 1 unsure, 1 declined	Hospital 4	
		GP practice 1	
		Needle exchange 1	
HMP Frankland (13)	8 tested	Prison 6	4
	2 applied, 3 declined	Hospital 1	
		Not stated 1	
HMP YOI Low Newton (5)	2 tested	Prison 2	1
	2 applied, 1 unknown		

* Some participants had multiple tests

Interviews identified personal and institutional barriers to the uptake of testing for HCV. Personal barriers related predominantly to lack of knowledge and fear of disease, a lack of awareness about the testing procedure, disease prognosis, treatment and outcome, and concern about confidentiality and stigma. Motivation was a personal factor that could facilitate uptake of testing. Institutional barriers related to the application to request a test, inadequate pre- and post- test discussion, and lack of continuity of care in the event of discharge or transfer from prison.

### 'Cause they're maybes scared, scared of knowing'

Ignorance and fear of disease was common amongst participants. Many were not aware of disease prognosis, treatment options and outcomes. Some assumed that a positive diagnosis was a 'death sentence' (See Geoff, Table 3).

**Table 3:** Personal barriers

GEOFF: Er, I don't know really, [pause] er, I don't really know, I mean, I think like I say, I think people are just frightened ye na [you know]. People are frightened to get the test ye na [you know], thinking that it could be a killer not knowing what, not knowing what it actually is, what it actually does to you, I mean?
ANDY: When I hear people on about it, it's, it kills you and that's it, (Uh hm) when you get it that's it. There's no way of getting rid of it, you've got it for life (Uh hm) and all the rest of it. Which, isn't quite true. I mean you can have it for the rest of your life, but you can clear it (Uh hm). There is like you say treatments available where you can get rid of it.
DANNY: Em, like I thought Hepatitis could live out of the body, certain Hepatitis's, but that some can't and some can. And the ones that I thought could, couldn't.. Things like that and I thought you could catch Hepatitis from like using towels and stuff but you can't.
EMMA(a): 'Cause I've got, there's no way I'm not having no, even if it was a one per cent chance of passing it down to me bairn [child] I wouldn't have it... Me bairn's done nowt [nothing], so it didn't ask to be born... to a junkie mother.
CHRISSIE: I had to, I had to do well most of it meself, with 'em I had to help because they couldn't get vein do you know what I mean? I had to have a go myself to try and get them something do you know?
TONY: I, the way I would think of it is more private. More privacy round it. Because there's a lot of people don't want to know they've got it because when you've got to put your sheets in and all that, you come down here with twenty, thirty other people and they're all what ye here for and all the rest of it.
EMMA(b): There was a lass with Hep C on Landing 2 and it was "Heppie" and that they call her, do you know what I mean? Yeah. Not very nice.
GARY: Well I know that it's contracted through blood ..., er it's very rare em and you can catch it through intercourse, sexual intercourse er, there's not many people that have, and the people that say that they have, I generally don't believe. I think they're just too embarrassed to say that they've been injecting drugs

HCV was often conflated or formed a constellation with other blood borne viruses such as HIV. There was confusion about mode of transmission and likely prognosis (See Andy, Table 3).

Perception of risk was mixed. Some prisoners attached great significance to sexual promiscuity whilst others, although recognising the dangers of sharing needles and syringes, did not always appreciate the risks involved in sharing other paraphernalia (See Danny, Table 3).

### 'You bear a responsibility not to pass it on'

The main motivations for testing were discussed in terms of social or personal responsibility. Some of those who had had the test were unaware of potential treatments but had undertaken the test to determine whether they needed to take action to avoid infecting others (See Emma(a), Table 3).

### 'They couldn't get any blood out of me'

Some prisoners complained that health care personnel lacked the skills required to take venous blood; although some acknowledged that the poor state of their veins contributed to poor venous access. Several prisoners had had to take their own blood because nursing or medical staff were unable to do so. (See Chrissie, Table 3)

Prisoners were not usually aware of how the test would be carried out, but this did not appear to be a barrier in itself. Most of those who were aware that testing would involve venepuncture felt that prisoners were not likely to be frightened of needles. Prisoners were not aware of the availability of a buccal swab or finger-prick blood-test.

### 'You don't want the whole wing to know'

There were concerns that confidentiality would not be maintained after accessing healthcare. These concerns were related to a fear of stigmatisation. Prisoners were concerned about the level of detail that they had to provide to apply for a test and about who might have access to these details (see Tony, Table 3).

The problem of stigmatisation was related to IDU and HCV infection (see Emma(b), Table 3). Prisoners were concerned that they may be stigmatised if there was a large bruise resulting from failed attempts at venepuncture, which would then be apparent to other prisoners and prison officers. Prisoners were more likely to attribute HCV infection to sexual promiscuity rather than to IDU (see Gary, Table 3).

### 'You have to put an app in. An app!!'

Prisoners were required to complete an application form ('app') for access to health care, including HCV testing. Many prisoners regarded the 'app' as unnecessarily bureaucratic and a barrier to testing in terms of a requirement to disclose IDU and the long waiting time (see Alison, Table 4). One prisoner had made three applications for a test and had not been tested.

**Table 4:** Institutional barriers

ALISON: That's the thing when you have to put all these applications in, (Ah ha) it puts you off.... It's because you, everything you do you've got, it's always put an application in, put an ap. in, and people are like "What, I have to put an app. in just to get to see if I've got a disease?" Do you know what I mean, that's the way people think. When you've got to put an app. in it's like I'll do it later, do you know what I mean? .... And it just, it's a nightmare, them apps. are definitely.
EMMA: I was tested... last year and I, I didn't have [Hep C], and I come back [to prison] and a certain doctor said that he would take bloods and everything. He took bloods off us and then I had to go and see him on Monday and he said "You've got Hepatitis virus in your body" and em, he said "You need more blood tests" and he took them, he took loads of blood off us and then he put these big stickers on saying what you call, infection and then diseases do you know what I mean, I felt stupid. And he says "Right, come and see us next Monday" and that was it. I was crying me eyes out. But he didn't, he didn't tell us nowt.
CHRISSIE: Yeah because like I'm, honest when I was rattling I was dead weak and I couldn't even remember I was just trying to get through my rattle. That's all I were thinking about.
JIMMY: they gave us a vaccination, then gave us another one, then I got me booster, but in the meantime while I was still using they wanted to take me blood afterwards to make sure if I had caught anything in the meantime (Yeah) but I ended up in prison (Right) so I couldn't get me blood taken.
COLIN: What I'm led to believe is we get the same as what you get in the community like you know so that's what we're told like. But er, I wouldn't know like I've been in a long time, but really I think, I don't think it's right. ... I think health care, I think you should have in, you know in an emergency then, in here you wait like you wouldn't in the community, but you wouldn't if it was an emergency in the community like you wouldn't wait like you know, and er, that's what I think.
DANNY: At the end of the day it's [Hep C] sort of, it's self inflicted isn't it? ... And we [IDUs] are just a society who, who people aren't really bothered about.
LS: Well, if you'd still been outside, would you have gone to your GP and asked for testing?
DANNY: Well I was, I did approach my GP and he referred us to the consultant but like I said it's been adjourned twice and I think that would've happened all the time, and they would've just got sick of us. ... Uh hm. They would've just got sick and said look, there's people there who really want it, wanting to do the treatment and you're just messing about by not staying off drugs.

### 'I think someone should be there to talk to you about it... he didn't tell us nowt [nothing]"

Although some prisoners reported that they had had the opportunity for pre- and post-test discussion with a healthcare professional, this was not always the case (see Emma, Table 4). In some cases test results were 'posted' to prisoners under their cell door without post-test information and discussion. Several prisoners, like Emma, compared the availability of 'counselling' with that provided before and after HIV testing and thought that the procedure was more robust for HIV than for HCV.

### 'I was just trying to get through my rattle [detox]. That's all I were thinking about'

The offer of testing opportunities may not arise at an appropriate time. Prisoners with short sentences or who are on remand (ie. awaiting trial) might not be able to keep their appointments for testing because of court appearances or early discharge. Some prisoners could not recall whether they had been offered a test because they had other priorities at first reception to prison or because they were suffering the effects of drug/alcohol withdrawal at the time (see Chrissie, Table 4).

### 'I got shipped out, so it never really happened'

Prisoners felt that there was not always adequate communication and transfer of information between prisons or between prisons and healthcare in the community. A prisoner, waiting for the test in one prison prior to transfer, would have to re-apply at the new prison. Two prisoners changed their minds about testing and had not reapplied after transfer as they would have to wait. Other prisoners were concerned that treatments or interventions initiated in the community would not be followed up in prison (see Jimmy, Table 4).

### 'in here you wait like you wouldn't in the community'

Some prisoners felt that they might not have the same access to healthcare for Hepatitis C whilst in prison as they would do in the community (see Colin, Table 4). Others believed that poor access to healthcare was encountered in prison and in the community because of stigma relating to IDU (see Danny, Table 4).

## Discussion

This project has identified personal and institutional barriers in participants' discourse that could explain the poor uptake of testing for HCV by prisoners. Personal barriers included: prisoners' fears and lack of knowledge about HCV, lack of awareness about the test procedure, and concerns about confidentiality and stigma. Institutional barriers included: the prisons' applications procedure for testing, inadequate pre- and post-test discussion, lack of appropriate approaches to offering testing, and lack of continuity of care on discharge and transfer.

There are many benefits in the early diagnosis of HCV infection: Knowledge of the status of HCV infection may reduce risk behaviour[11,12]; anti-viral treatment can eradicate infection in 40–85% of cases depending on viral genotype[13]; secondary preventive measures, such as reducing alcohol intake, can reduce the risk of developing hepatocellular carcinoma[14]; and the need for other health protection interventions, such as Hepatitis B and Hepatitis A vaccination may be identified. Yet, the majority of those with HCV infection remain undiagnosed; in England, fewer than 20% of those infected are diagnosed[1].

This study is not unique in identifying a poor understanding of HCV infection amongst IDUs[10]. It is possible that increasing the awareness of the disease and the benefits of diagnosis will increase the uptake of testing.

We know that the uptake of testing for HCV by prisoners is low and that there is a higher prevalence of HCV amongst the prison population because of the higher proportion of injecting drug users in the custodial environment. This study demonstrates that: personal and institutional barriers may adversely affect the uptake of testing for HCV in prisons; there is general lack of awareness and fear of HCV amongst prisoners with a history of injecting drug use; prisoners are concerned about the potential for breaches in clinical confidentiality and about the stigma associated with a diagnosis of HCV. These findings will enable development of interventions to improve the uptake of testing for HCV in prisons and to develop care pathways that provide seamless healthcare within and outside the custodial setting.

### Limitations of this study

Participants were recruited from three prisons with representation from male and female prisoners, and long-stay and short-stay establishments. However, only five women prisoners were interviewed, none from long-stay prisons. Although there is a potential for bias because data saturation was not reached amongst female participants, nevertheless, the interviews elicited a wide spectrum of opinion and experience. The presence of CARATs worker may have influenced the content and level of detail of the interviews. However, it appeared that prisoners were comfortable with the presence of the CARATs worker and in most cases appeared to have already established a degree of rapport with either an individual officer or the CARATs team in general.

One key limit to the study was that we were unable, within existing resources, to also interview prison personnel and health professionals working in the prisons to ascertain their views of barriers to uptake of HCV testing. We are therefore not able to identify potential disincentives for carrying out testing, for example the impact on resources of the potential added burden of care and treatment for those diagnosed with HCV. Nonetheless, this study is an example of effective collaborative working between multiple stakeholder organisations. A multidisciplinary steering group facilitated the recruitment of participants and conduct of the project.

## Conclusion

This study has identified some factors that may contribute to the poor uptake of HCV testing in prisons. There is a need to develop approaches to increase the uptake of testing by raising awareness amongst prisoners about HCV infection, optimising testing pathways that support appropriate testing at appropriate times during a prisoner's stay in prison, ensuring adequate pre- and post-test discussion, and by developing care pathways for HCV that enable seamless continuity of care.

## Competing interests

The author(s) declare that they have no competing interests.

## Authors' contributions

LS carried out the interviews of participants; all authors contributed to the design of the study, data analysis and drafting of the manuscript; all authors read and approved the final manuscript.

## Pre-publication history

The pre-publication history for this paper can be accessed here:


